# *Coxiella burnetii* (Q fever) exposure in recycling, non-recycling workers and dogs in a major urban area of Brazil

**DOI:** 10.3389/fpubh.2026.1820795

**Published:** 2026-06-08

**Authors:** Altina Bruna de Souza Barbosa, Danilo Alves França, Cassiana Dahlke Machado, Igor Silva Silito, Louise Bach Kmetiuk, Marcelo B. Labruna, Alexander Welker Biondo

**Affiliations:** 1 Graduate College of Cell and Molecular Biology and Department of Veterinary Medicine, Federal University of Paraná (UFPR), Curitiba, Brazil; 2 Department of Preventive Veterinary Medicine and Animal Health, School of Veterinary Medicine and Animals Science, University of São Paulo (USP), São Paulo, Brazil; 3 Graduate College of Veterinary Science, Federal University of Paraná (UFPR), Curitiba, Brazil; 4 Center of Epidemiology, City Secretary of Health, Curitiba, Brazil

**Keywords:** environmental transmission, occupational risks, one health, Q fever, vulnerable populations

## Abstract

**Introduction:**

Q fever has been considered a worldwide zoonosis caused by Coxiella burnetii, mostly transmitted by respiratory routes in contaminated environments with bio logical waste. Although urban recycling facilities may concentrate contaminated materials, their significance as potential environmental sources for C. burnetii infection has not been investigated to date.

**Methods:**

Accordingly, the aim of this study was to assess previous exposure to C. burnetii in recycling, non-recycling workers and their dogs from Curitiba, a major urban area currently ranked 8th in population with 1.8 million habitants, 7th in Gross Domestic Product (GDP), and 10th in Human Development Index (HDI) out of 5,569 Brazilian cities.

**Results:**

In overall, 6/278 (2.15%) workers and 7/137 (5.1%) dogs were seropositive to C. burnetii by indirect immunofluorescence assay (IFA).

**Discussion:**

Due to low seropositivity, no risk factor was statistically associated with C. burnetii exposure. This was the first study to investigate C. burnetii infection in workers and dogs of urban recycling centers. Although low, positive cases have suggested likelihood of environmental expo sure to Q fever. Such findings highlight the importance of future investigations, as well as the need to ensure proper use of respiratory protective equipment among recycling workers in major urban areas of Brazil and worldwide.

## 1 Introduction

Q fever has been considered a worldwide distributed zoonosis caused by the intracellular bacterium *Coxiella burnetii*, with a wide range of human, domestic and wildlife animal hosts (1). Infection primarily occurs through the inhalation of contaminated particles present in dust, secretions, or animal waste, and may lead to acute or chronic disease, including hepatitis, pneumonia, and endocarditis. Although disease may potentially impact public health, especially among exposed individuals to contaminated environments, Q fever remains a neglected disease, with limited surveillance, underdiagnosis, misdiagnosis, and a scarcity of studies in vulnerable populations, particularly in urban areas worldwide (2).

Airborne transmission of *C. burnetii* has historically been associated with direct contact with domestic animals during parturition, especially through the inhalation of particles from placental tissues and birth fluids (3). Also, a systematic review has described dust, air, soil and liquids contamination in 28 Q fever outbreaks in endemic settings. Environmental contamination was most commonly associated with ruminant livestock populations, despite geographically limited (4).

Recycling material workers represent a particularly vulnerable population to neglected infectious agents due to the nature of their occupational activities (5–7). These workers have direct and continuous contact with urban waste, often without adequate use of personal protective equipment, handling potentially contaminated materials such as paper, fabric, straw, animal-derived packaging, and organic remains in various stages of decomposition (8). In addition, many workers own semi-domiciled dogs with access to streets and outdoor garbage, which may facilitate the introduction and persistence of zoonotic agents in both household and workplace environments (9).

Although recycling centers in urban areas may potentially concentrate contaminated materials, no *C. burnetii* serosurvey has been conducted in recycling material workers to date. Accordingly, the aim of this study was to assess *C. burnetii* exposure and potential associated risk factors in recycling, non-recycling workers and their dogs from a major metropolitan area in Southern Brazil.

## 2 Materials and methods

### 2.1 Ethical statement

The study herein was approved by the National Human Research Ethics Committee (protocol number 68636123.3.0000.0102, report number 6.113.318/2023) and by the Animal Use Ethics Committee (protocol number 009/2023) of the Federal University of Paraná.

### 2.2 Study area

The present study was conducted in Curitiba (25°25′47“S, 49°16′19” W), State capital of Paraná and a major urban area currently ranked 8th in population with 1.8 million habitants, 7th in Gross Domestic Product (GDP), and 10th in Human Development Index (HDI) with 0.823 (very high), out of 5,569 Brazilian cities. In addition, Curitiba has been indicated as the most sustainable city in Brazil and Latin America, ranked among the top 20 sustainable cities worldwide (10).

Curitiba has been also recognized nationwide as highly effective in recycling rate and waste management, pioneering and serving as an international reference in selective waste collection. Curitiba currently has contracts with 49 recycling associations throughout the city, each warehouse employing an average of 15 workers. Out of these associations, 11 are located in the Parolin neighborhood, which also presents a significant concentration of informal recycling workers. Thus, recycling workers and residents of the Parolin neighborhood were included in the study, given their shared exposure to the same environment characterized by a high concentration of urban solid waste within the recycling warehouses.

A calculated minimum sample size of 278 (277.5) participants was obtained to assess the human seropositivity rate for *C. burnetii*, assuming an absolute precision of ±5% and an approximate population of 900 to 1,000 recycling workers officially registered in Curitiba city. The calculation was based on the Wald method, with a 95% confidence level and an expected seropositivity rate of approximately 50%, since the local prevalence was unknown at the time. The estimate was performed using the epiR package in RStudio v.2025.05.0 (11). Participants were selected through a non-probabilistic convenience sampling method. Recruitment was conducted through active invitations at recycler associations and among residents of the Parolin neighborhood, targeting individuals with similar environmental exposure. Dog samples were obtained by convenience, with the signed consent and participation of their respective owners.

### 2.3 Sample collection

Human blood samples were collected by cephalic vein puncture, with procedures performed by certified physicians and nurses at local Primary Health Care Units. Dog blood samples were obtained via jugular venipuncture performed by certified veterinarians, after the owner signed a consent form. All blood samples were placed in sterile tubes containing a serum-separating gel without anticoagulant, centrifuged at 1,500 *g* for 5 min, and the serum was stored at −80 °C until processing. After samplings, as ethical research counterpart, dogs were given anti-rabies and multiple (species-specific) vaccines, deworming treatment and antiflea products. All interventions were performed by certified veterinarians, free-of-charge, registered in pet vaccination certificates and given to owners.

### 2.4 Epidemiological data

A standardized epidemiological questionnaire was administered to all human participants to assess associated potential risk factors with previous *C. burnetii* exposure (Supplementary Table 1). The survey included questions on demographic characteristics (such as sex and age), occupational habits, use of personal protective equipment (PPE), history of contact with animals (either domestic or livestock), consumption of raw meat, recent history of fever or symptoms compatible with Q fever, among other variables related to lifestyle and environmental conditions. The collected information was used for statistical analysis of factors associated with seropositivity.

To identify a history of food insecurity, the epidemiological questionnaire included questions based on Food Insecurity Screening Tool (TRIA). TRIA is a validated instrument specifically developed to identify the risk of food insecurity, rather than providing a graded clinical diagnosis, as performed by the Brazilian Food Insecurity Scale (EBIA) (12, 13). The TRIA consists of two objective questions (Yes/No): (1) ‘In the last 3 months, did food supplies run out before the family had money to buy more?’ and (2) ‘In the last 3 months, did you have to eat only certain types of food because the money ran out?’. Since this study performed IgG class antibody detection for tracking past contact with the pathogen, the questions were adapted to include any period beyond the initial 90 days. Following the instrument’s protocol, participants were classified as ‘positive’ for risk of food insecurity if they gave an affirmative answer (Yes) to at least one of the two questions.

### 2.5 Serological testing

Human serum samples were analyzed using an indirect immunofluorescence assay (IFA) that was internally developed and validated, as previously described (14, 15). The assay employed the *C. burnetii* strain At12, maintained through successive passages in Vero cell monolayers. Serum samples were initially screened at a 1:64 dilution using fluorescein isothiocyanate (FITC)-conjugated anti-human IgG antibodies. Previously characterized positive and negative control sera were included as internal controls in each assay run. Samples exhibiting specific fluorescence were titrated by twofold serial dilutions, with the endpoint titer defined as the highest dilution showing distinct fluorescence (16). The dog serum samples were tested using the same in-house protocol, except for the use of fluorescein isothiocyanate (FITC) IgG anti-dog antibody (Sigma, St. Louis, MO, USA).

### 2.6 Statistical analysis

Statistical analyses were performed using the R software version 4.5.2 (R Core Team, 2024) (17), which was integrated into the RStudio development interface, version 2026.01.0 + 392 (18). Assessment of association between *Coxiella burnetii* seropositivity and the independent variables was obtained by Fisher’s Exact Test, considered the most appropriate method for contingency tables with low expected frequencies in individual cells (counts < 5). The magnitude of these associations was estimated using the Odds Ratio (OR) with a 95% Confidence Interval (CI). For each categorical variable, a reference group (OR = 1.00) was established, typically representing the category with the lowest hypothesized exposure risk. Statistical significance was defined at level of *p* < 0.05. Due to the limited number of reactive samples, variables with zero frequency in the positive group were marked as “not applicable” (N/A) for OR estimation.

## 3 Results

A total of 278 samples were collected, comprising 178 from recycling and 100 from non-recycling workers, all residing in the same Parolin neighborhood, characterized by a high density of recycling warehouses, an environment associated with high exposure to urban solid waste. The overall seropositivity of anti-*C. burnetii* antibodies in the sampled human population was 2.15% (6/278), with endpoint titers ranging from 64 to 128. Among dogs, 7/137 (5.1%) were seropositive, with antibody titers ranging from 64 to 256.

The distribution of seropositivity across occupational categories was 1.7% (3/178) for recycling and 3.0% (3/100) for non-recycling workers (OR = 1.77; 95% CI: 0.38–8.16). Due to the low number of seropositive events, no variables reached statistical significance (*p* > 0.05), and all 95% confidence intervals encompassed the null value (OR = 1.0) (Table 1).

**Table 1:** Associated risk factors for *C. burnetii* serosurvey in recycling and non-recycling workers of Curitiba City, Paraná State, Brazil.

Variable	Category	Positive (n)	Negative (n)	Total (n)	% Positive	*p*-value	OR (95% CI)
Occupation	Non-recycling	3	97	100	3.0	0.670	1.00
	Recycling	3	175	178	1.7		0.56 (0.07–4.23)
Gender	Female	4	176	180	2.2	1.000	1.00
	Male	2	96	98	2.0		0.92 (0.08–6.53)
Food insecurity	No	2	121	123	1.6	0.697	1.00
	Yes	4	151	155	2.6		1.6 (0.23–17.96)
Food hygiene	Chlorine solution	2	55	57	3.5	0.894	1.00
	Only water	3	156	159	1.9		0.53 (0.06–6.51)
	Does not wash	0	18	18	0.0		0 (0–17.09)
	Other products (soap/vinegar)	1	43	44	2.3		0.64 (0.01–12.72)
Raw meat consumption	No	4	150	154	2.6	0.695	1.00
	Yes	2	122	124	1.6		0.62 (0.05–4.38)
Manual soil manipulation	No	2	182	184	1.1	0.184	1.00
	Yes	4	90	94	4.3		4.02 (0.56–45.25)
Dog ownership	No	1	68	69	1.4	1.000	1.00
	Yes	5	204	209	2.4		1.66 (0.18–79.95)
Cat ownership	No	4	188	192	2.1		1.00
	Yes	2	84	86	2.3		1.12 (0.1–7.98)
Education level	Up to Complete Elementary Education	2	189	191	1.0	0.079	1.00
	Completed High School or Higher Education	4	83	87	4.6		4.53 (0.63–50.95)

Due to low seropositivity, no association risk factor was statistically significant.

In the present study, due to the absence of statistical significance (*p* > 0.05) and with extremely wide 95% CIs for ORs, formulating categorical hypotheses about transmission routes is speculative. Some observations for future studies were following reported. Specifically, 66.7% (4/6) of the seropositive individuals reported a history of food insecurity. Furthermore, manual soil manipulation was reported by 83.3% (5/6) of positive individuals, with only one seropositive respondent (Human 05) denying this practice. These data underscore the significance of soil as a critical environmental reservoir for *C. burnetii* spore-like variants, particularly in recycling-dense hubs where the ground may be impregnated with pathogen-laden dust. The seropositive individuals reported a heterogeneous educational background, ranging from incomplete primary education to university degrees. In the context of IgG-class antibody detection indicates historical exposure the geographical provenance of seropositive individuals remains an essential interpretive element in mapping the pathogen’s regional spread. This demonstrated that while 50.0% (3/6) of cases were of individuals from Curitiba, reactivity was also identified in individuals from Campo Mourão, Assaí, and Ponta Grossa. Regarding the canine-human interface, only one reactive dog (Dog 02) shared a positive serological status with its owner. The frequent asynchrony in seropositivity, even with high titrations in dogs (Dog 03 and Dog 07) and negative owners, suggests that transmission is predominantly mediated by environmental bioaerosols or dust rather than direct interspecies contact. This reinforces the hypothesis that both species are being independently exposed to a shared environmental source within these recycling clusters (Supplementary Table 2).

## 4 Discussion

The *C. burnetii* seropositivity observed herein (2.15%) was low, particularly when compared to previous surveys conducted in populations traditionally considered at risk, such as rural workers, animal health professionals, and other exposed groups investigated in the same Brazilian region. For example, a serosurvey in quilombola communities of Paraná State reported a human prevalence of 22.0% (44/200) and dog of 5.0% (1/20), possibly associated with frequent contact with rural environments and domestic livestock (15). Similarly, a survey conducted among febrile patients in São Paulo found a seroprevalence of 21% (19), involving people experiencing homelessness reported a rate of 14.8% (30/203) (20).

On the other hand, very low or near-zero prevalence rates have also been observed in vulnerable populations with limited contact with traditional sources of infection, such as Indigenous communities with human prevalence of 0.90% (8/893) and dog of 2.04% (21) and residents of oceanic islands with human prevalence of 1.49% (5/335) and dog of 0% (0/352) (22). These findings reinforced that exposure to Q fever may be highly dependent on environmental context and the nature of the individual interaction with the environment.

The identification of seropositive individuals suggested multiple infection pathways within the recycling ecosystem. Beyond the occupational hazard of inhaling contaminated dust during waste processing (23), the environmental stability of *C. burnetii* allows for broader dissemination. The suspension of bioaerosols from stored organic materials in enclosed spaces represented a risk not only to workers but also to nearby residents (24). In this context, the airborne dispersal of the pathogen from high-density recycling hubs may have acted as a primary transmission route for the local population, regardless of direct occupational involvement.

Additionally, the presence of dogs and other animals circulating within or around recycling centers may pose an additional exposure source, since secretions, urine, or feces from infected animals can contaminate the environment (25). Therefore, although direct contact with animals during parturition may not be part of these workers’ routine, the possibility of indirect environmental exposure, particularly through aerosols from long-contaminated waste (3, 23), cannot be ruled out. This hypothesis was further supported by reports of Q fever outbreaks occurring in non-conventional settings, including environments without direct livestock contact, where airborne dispersion and environmental contamination were implicated as primary transmission routes (14, 23, 26–29).

In the present study, the indirect immunofluorescence assay (IFA) was employed exclusively to assess previous exposure to *C. burnetii*, rather than to differentiate between acute and chronic infection, since all individuals were asymptomatic at the time of sampling. The antigen used consisted of an in-house preparation, capable of detecting antibodies against *C. burnetii* without distinction between antigenic phases. This approach has been considered reportedly appropriate for non-clinical seroepidemiological surveys (14).

Despite the growing recognition of Q fever as a relevant zoonosis across continents, most surveillance and research efforts remain focused on rural areas and populations traditionally associated with livestock farming, such as farmers, veterinarians, and slaughterhouse workers (30). In urban settings, particularly among marginalized populations such as waste pickers, the disease has remained epidemiologically and institutionally neglected. Although Q fever outbreaks and transmission have been documented in waste-related environments (27), most surveillance and research efforts has continuously focused on traditional high-risk groups, such as farmers, veterinarians, and slaughterhouse workers (30). This disparity highlighted a potential gap in surveillance and research involving populations exposed to urban waste.

Although seropositivity for *C. burnetii* among recycling workers and residents in high-density recycling facility clusters was low, the six positive cases indicate that exposure to the agent is plausible and may be related to frequent contact with potentially contaminated recyclable materials. This study represents a preliminary finding and the first investigation specifically focused on *C. burnetii* infection in recycling workers and community residents from areas with a high concentration of recycling warehouses. The results highlight the need for further research with an environmental focus. Q fever, although rarely recognized in urban environments, should be incorporated into surveillance and prevention strategies targeting workers exposed to solid waste.

As limitation of the present study, although the in-house IFA protocol applied herein was adapted by modifying only the secondary antibody, this assay was not species-specific validated for dog samples. Despite widely and routinely used, the results herein should be cautiously interpreted, as the precise performance in dogs was not fully established. As samplings were conducted only in the Parolin neighborhood, total local population of recycling workers was much smaller and indicated a conservative oversized sampling, completed by informal recycling workers and exposed residents. Also, provision of free vaccination, deworming and antiflea as an “ethical counterpart” may have introduced a selection bias, as participant owners could be more likely interested in such free-of-charge veterinary services, with specific concerns or with higher interaction with their dogs, which may have influenced in the lower frequency of *C. burnetii* antibodies observed herein.

## Data Availability

The original contributions presented in the study are included in the article/Supplementary material, further inquiries can be directed to the corresponding author/s.
