# Supplementary material for: Coxiella burnetii (Q fever) exposure in recycling, non-recycling workers and dogs in a major urban area of Brazil
Source: Front Public Health. 2026 Jun 8;14:1820795. doi: 10.3389/fpubh.2026.1820795 (PMC13284059; doi:10.3389/fpubh.2026.1820795)
Supplement: Supplementary file 1 [file Data_Sheet_1.PDF]

Supplementary Material

Supplementary Table 1. Epidemiological questionnaire applied to recycling and non- recycling workers in a major urban area of Brazil.

---

1. Full Name:____
2. Is registered in the CadÚnico (Single Registry for Social Programs)? ( ) Yes ( ) No.
   •
3. Do you receive any government assistance? ____________________
4. Phone number:
   •
5. CPF (Brazilian Social Security Number):
6. Date of birth:____/____/____.
7. Gender:
8. Home address:
9. Marital status: ( ) single ( ) married ( ) widow ( ) common-law marriage ( ) divorced ( ) other
10. Ethnicity: ( ) White ( ) Black ( ) Brown ( ) Indigenous ( ) Other_______________
11. How many people live in the house?
12. Average Family income:
13. Did food supplies ever run out before the family had money to buy more, or have you ever had financial difficulties buying food?
14. Level of education

( ) No formal education	( ) No formal education	( ) No formal education
( ) Incomplete Primary (1st–4th grade)	( ) Incomplete Primary (1st–4th grade)	( ) Incomplete Primary (1st–4th grade)
( ) Complete Primary	( ) Complete Primary	( ) Complete Primary

15. City of Origin: _________________________________
16. Health Clinic Reference: Do you have a specific health unit you go to? ( ) Yes. Which one? ________________ ( ) No
17. Vaccinations: Are your vaccinations up to date? ( ) Yes ( ) No
18. Travel: Do you usually travel to other municipalities? ( ) No ( ) Yes. To which cities? ________________
19. Do you work with recycling? ( ) Yes ( ) No (If no, skip to question 34)
    - How long have you worked in this field? ________________
20. Type of work: Do you work in collection, sorting, or both? ________________
21. Materials sold: ( ) Plastic ( ) Cardboard ( ) Aluminum ( ) Glass ( ) Other: __________

22. Sales & Organization: Where do you sell the collected materials? _______________
    - Are you a member of a recycling cooperative or are you self-employed? ( ) Association/Cooperative: ________________
23. Do you use Personal Protective Equipment (gloves, mask, boots)? ( ) No ( ) Yes.
    - Which ones? ________________
24. Have you ever had an accident with sharp materials while working? ( ) No ( ) Yes.
    - With what material? ________________
25. Storage Location: After collection, where are materials stored?
    - ( ) Residence ( ) Warehouse ( ) Other: _______________
26. Storage Conditions: Is the storage area covered? ( ) Yes ( ) No
27. How long does the material remain stored? ________________
28. How is the material stored? ( ) Large bags ( ) Plastic bags ( ) Boxes ( ) Other: __________
29. Where is the waste from non-marketable materials discarded? ________________
30. Does flooding occur after rain where materials are stored? ( ) Yes ( ) No
31. Do you see bats where materials are stored? ( ) Yes ( ) No
32. Do you see rats where materials are stored? ( ) Yes ( ) No

If yes (rats): What time? ( ) Day ( ) Night ( ) Both

Frequency: ( ) Every day ( ) Once a week ( ) More than once a week ( ) I don't see them

33. Does flooding occur at your home after rain? ( ) Yes ( ) No
34. Do you see bats at home? ( ) Yes ( ) No
35. Do you see rats at home? ( ) Yes ( ) No
    - If yes (rats): What time? ( ) Day ( ) Night ( ) Both
    - Frequency: ( ) Every day ( ) Once a week ( ) More than once a week ( ) I don't see them
36. Have you ever been bitten by a rat? ( ) Yes ( ) No
37. Water Source: ( ) Spring ( ) Well ( ) Treated piped water
    - Water Tank: Do you have a water tank? ( ) Yes ( ) No
38. Do you have a home or community garden? ( ) Yes ( ) No

- If yes: What is the irrigation source? ________________

39. Do you usually wash fruits and vegetables before meals? ( ) Yes ( ) No
40. If yes: How do you wash them? ( ) Only water ( ) Water and soap/vinegar ( ) Chlorine solution
41. Do you usually wash your hands before meals? ( ) Yes ( ) No
42. Do you have the habit of eating raw or undercooked meat? ( ) No ( ) Yes. Which species? _______________
43. Do you have direct contact with soil/earth? ( ) Yes ( ) No
44. Do you have the habit of biting your nails? ( ) Yes ( ) No
45. Pets: Do you have animals? ( ) No ( ) Yes. How many and which species? _____________
46. Are the animals vaccinated? ( ) Yes: __________ ( ) No ( ) Does not have animals
47. Do the animals go with you during recycling collections? ( ) Yes ( ) No ( ) Does not have animals
48. How are collections performed? ( ) Handcart ( ) Other: ________________
49. Do you have children? ( ) No ( ) Yes. What are their ages? ( ) Adult ( ) Child
50. If you have been pregnant, have you ever had a miscarriage? ( ) No ( ) Yes. How many? __
51. Do your children work in recycling? ( ) No ( ) Yes. Weekly frequency: __________
52. Do you have any health problems? ( ) No ( ) Yes. Which? _________________
53. Have you ever been bitten by a parasite (tick, flea, head louse, or body louse)? ( ) Yes ( ) No

---

Supplementary Table 2. Individual results of seropositive samples for the *C. burnetii* serosurvey in recycling, non- recycling workers and dogs in Curitiba City, Paraná State, Brazil.

Samples	Description
Human 01	Endpoint titer 128; 45 years old, non-recycler, female, previous experience of food insecurity, educational level – bachelor's degree; home city – Curitiba; life-long resident of the study area; does not eat raw meat; manual soil manipulation – yes; owned 2 dogs.
Human 02	Endpoint titer 128; 50 years old, recycler, male, previous experience of food insecurity, educational level – incomplete primary education home city – Campo Mourão (PR); living for 27 years in the study area; does not eat raw meat; manual soil manipulation – yes; 1 dog.

Human 03	Endpoint titer 64; 66 years old, non-recycler, female, previous experience of food insecurity, educational level – incomplete primary education; home city – Assaí (PR); resident of Parolin for 20 years; doesn't eat raw meat; manual soil manipulation – yes; 3 dogs.
Human 04	Endpoint titer 128; 54 years old, non-recycler, male, no previous experience of food insecurity, educational level – complete high school; home city – Ponta Grossa (PR); resident of the study area for 34 years; doesn't eat raw meat; manual soil manipulation – yes; 1 dog.
Human 05	Endpoint titer 64; 22 years old, recycler, female, no previous experience of food insecurity, educational level – incomplete bachelor's; home city – Curitiba (PR); life-long resident of the study area; eat raw meat (cattle, fish); manual soil manipulation – no; 3 cats.
Human 06	Endpoint titer 128; 40 years old, recycler, female, previous experience of food insecurity, educational level – complete high school; home city Curitiba (PR); life-long resident of the study area; eat raw meat (cattle); manual soil manipulation – yes; 1 dog and 1 cat.
Dog 01	Endpoint titer 64; negative non-recycler owner.
Dog 02	Endpoint titer 128; positive non-recycler owner.
Dog 03	Endpoint titer 256; negative non-recycler owner.
Dog 04	Endpoint titer 128; negative non-recycler owner.
Dog 05	Endpoint titer 64; negative non-recycler owner.
Dog 06	Endpoint titer 64; negative recycler owner.
Dog 07	Endpoint titer 256; negative recycler owner.
